# Mitigating Issues With/of/for True Personalization

**DOI:** 10.3389/frai.2022.844817

**Published:** 2022-04-26

**Authors:** Harri Oinas-Kukkonen, Sami Pohjolainen, Eunice Agyei

**Affiliations:** Oulu Advanced Research on Service and Information Systems, University of Oulu, Oulu, Finland

**Keywords:** personalization, tailoring, customization, persuasive systems, change management

## Abstract

A common but false perception persists about the level and type of personalization in the offerings of contemporary software, information systems, and services, known as Personalization Myopia: this involves a tendency for researchers to think that there are many more personalized services than there genuinely are, for the general audience to think that they are offered personalized services when they really are not, and for practitioners to have a mistaken idea of what makes a service personalized. And yet in an era, which mashes up large amounts of data, business analytics, deep learning, and persuasive systems, true personalization is a most promising approach for innovating and developing new types of systems and services—including support for behavior change. The potential of true personalization is elaborated in this article, especially with regards to persuasive software features and the oft-neglected fact that users change over time.

## Introduction

During the past few decades many contributions have been made to the body of scientific knowledge on personalized information technology, especially regarding user-modeling and user-adapted interaction (e.g., Brusilovsky, 2001; Fischer, 2001; Kobsa, 2001). The general audience has been awakening to this topic little by little since the late 90s after the introduction of e-commerce services for consumers. More recently requests for data analytics and adaptation of services to user needs have rapidly grown, and myriads of web-based and mobile services now claim to offer personalized solutions (Langrial et al., 2012).

Personalization as a research construct is, however, much more complex than it appears on the surface (Tam and Ho, 2005, 2006). Moreover, a common but false perception about the level and types of personalization, known as the *Personalization Myopia* (Oinas-Kukkonen, 2018) persists: researchers tend to think that there are many more personalized services than there really are. Likewise, the general audiences are under the impression that they are offered personalized services when it is not the case, and practitioners often have a mistaken idea of what actually makes a service or system personalized (Oinas-Kukkonen, 2018).

In this article, we will discuss personalization myopia and especially how to undo its consequences and thus ultimately to do away with it. The fundamental question under investigation is how to draw advantages from *true personalization* when designing such systems. After describing personalization myopia, two issues with strong personalization are recognized: going beyond mere personalized content by offering personalized software features and addressing the oft-neglected fact in personalization efforts that also users change over time. Finally, it is explained how artificial intellegence techniques can be employed to improve true personalization and ethical considerations for true personalization are discussed.

## Types of Personalization

The depth and actuality of personalization implementations vary. For this reason, Oinas-Kukkonen (2018) suggests a taxonomy of personalization instantiations in which true and false personalization are differentiated and the depth of true personalization in systems and services is suggested to vary between low and high levels. The latter archetypes are called *weak* and *strong personalization*. See Table 1.

**Table 1:** Depth and reality of personalization (Oinas-Kukkonen, 2018).

**High-level**	Fake	Strong
**Low-level**	Fake	Weak
	**Fake**	**True**

Contemporary web and mobile users have become accustomed to services, which actively use a user's name in feedback sent to the user. However, in many if not most of the cases where service providers claim to offer personalized services, they may be just trying to make the user feel more comfortable without offering personalized features other than using one's name; or they are simply unknowledgeable of what personalization really would involve. This is *fake personalization*, in other words, it is actually not personalization at all. One reason why this has become so popular among commercial services is that consumers seem to be influenced by this approach in spite of it not being true personalization. This is because how people *perceive* the services to be rather than what the services actually are, which persuades people to take action.

Sometimes users may think that they are provided with information that is personalized for them individually, whereas in reality these systems offer information that is only slightly modified from standard information or is in truth targeted at a larger group of users. Targeting at a given user segment is known as *tailoring* (Oinas-Kukkonen and Harjumaa, 2009), and it is a widely studied software feature (Torning and Oinas-Kukkonen, 2009). We suggest that tailoring is low-level or *weak personalization*, which naturally may be valuable even if it is not the more sophisticated form of true personalization. Information provided by the system may indeed be persuasive if it is tailored to the potential needs, interests, use context, or other factors relevant to a user group. For example, Parmar et al. (2009) studied the use of weak personalization through a tailored health solution designed to influence the health behaviors of rural Indian women, aiming at increasing their awareness about menses and maternal health. The system employed social cues to increase this group's, i.e., rural women, perceived behavioral control and motivation to challenge existing social beliefs and practices, and in this manner persuading them to follow evidence-based health practices. Their study demonstrates how weak personalization, in this setting through providing tailored health content for this particular group, rather than providing either generic or individualized health content can be useful. In other studies, the impact of computer-tailored health interventions on behavior change were investigated and it was concluded that that tailored interventions should adapt not only the content of the message but also how the message is presented to the users (van Genugten et al., 2012; Nikoloudakis et al., 2018).

True high-level personalization, *strong personalization*, would mean that the information system really offers individualized content and/or services for its users. For instance, a system would first provide arguments that are most likely to be relevant for the individual user before any wider pre-defined user group or instead of simply presenting them random order. For an example of the effectiveness of strong personalization, Andrews (2012) investigated how a user's degree of extraversion influences perceived persuasiveness and perceived trustworthiness of a system. According to this study dependencies between a user's personality and perception of the system were evident. In another example, Dijkstra (2006) studied the impact of persuasive messages on students who smoked tobacco daily. After completing a pre-test questionnaire on a computer, the participants read information about their own condition and filled in an immediate post-test questionnaire. After 4 months, they were sent a follow-up questionnaire to assess their quitting activity. The results showed that significantly more participants quit smoking after 4 months when they received personalized feedback instead of standardized information. Moreover, the effect of condition on quitting activity was mediated by individuals' evaluations of the extent to which the information took into account personal characteristics.

Naturally, strong and weak personalization approaches are closely related and demonstrate the same spirit of developing information systems and services, and in some cases, they can even co-exist. Yet, tailoring as a weak approach is only low-level personalization. What tailoring may offer is often mistakenly considered by end-users as the pinnacle of what personalization can offer, which may give a false idea about the potential of personalization and in this manner contribute to the prevalence of the Personalization Myopia (Oinas-Kukkonen, 2018). Another reason why this Myopia is so widespread today and is likely to persist in the near future is that strong personalization does demand exceptionally careful modeling and analysis of the individual user and his or her susceptibility (Andrews, 2012; Kaptein et al., 2012) for information presentation and perhaps the adaptation of software functionalities offered. Modeling this is a most intriguing but challenging task, requiring a mindset and often also time and data analysis resources of an academic researcher, which are not necessarily available to all practitioners. There is a difference also between customization and personalization. *Customization* means the modification of the system and/or its preferences *by* the user. Thus, customization may be considered as a form of personalization. An example of a combination of customization and personalization would be software that lets a user define when he or she wants to be reminded.

To help understand what is required when designing weak vs. strong personalization, the Persuasive Systems Design model's Use and User Contexts can be applied (Oinas-Kukkonen and Harjumaa, 2009). **Use Context** considers characteristics arising from the problem domain and potential user segments in it, whereas **User Context** aims at recognizing individual differences. In this sense, weak personalization is mostly linked with *Use Context*, whereas strong personalization is inherently linked also with *User Context*. In strong personalization, an individual's User Context such as a user's susceptibility to the content presentation strategy at hand needs to be thoroughly understood, which may require, for example, understanding one's need for cognition (Petty and Wegener, 1998; Cacioppo et al., 2010), stage of change (Sporakowski et al., 1986), and integration into the individual life situation, experience, and self-efficacy (Borghouts et al., 2021), or perhaps even one's personality (Halko and Kientz, 2010) or temperament dimensions that impact affective-motivational and attentional systems that includes scales such as satisfaction/frustration, attention shifting/focusing, sociability, pleasure reactivity, discomfort, fear, and activity level (Rothbart and Bates, 1977; Rothbart et al., 2000; Evans and Rothbart, 2007; Rothbart, 2007). The need for modeling user susceptibility is especially important when designing for health behavior change support systems (Oinas-Kukkonen, 2013) that seek to offer true personalization (Berkovsky et al., 2012). An important approach specific to personalized persuasive systems has been suggested (Kaptein and Eckles, 2010), known as persuasion profiling, which is a collection of expected effects of different influence strategies for a specific individual. In spite of the complexity of modeling User Context and temptation of focusing only on user perceptions instead of actual outcomes, strong personalization still is a most promising approach to innovation, as well as for supporting behavior change in important areas such as health and sustainability (Berkovsky et al., 2012; Oinas-Kukkonen, 2013).

Even if there are substantial amounts of research on personalization, the volume of implemented software applications offering strong personalization is not as notable—and at the very least it is less notable than academics seem to think (Oinas-Kukkonen, 2018). Moreover, sometimes there seems to be much more interest in weak personalization rather than strong personalization, which, of course, is not to be automatically deemed as “bad”. Indeed, a separation between weak and strong personalization does not put forward the claim that the weak would be worse than the strong. Rather both weak and strong personalization approaches are needed just as both weak and strong ties are in social network analysis. At the end of the day, it seems to be oftentimes the perception of personalization rather than the actual, which persuades people into action.

## Issues With Strong Personalization

Personalization is often only considered regarding contents provided for the users. For instance, when a user sets personal goals and tracks parameters that are important to him or her, the system may provide progress feedback and/or suggestions based on user preferences. Thus, the contents received by a user are individualized, but the software functionality and features remain the same for all users. Similarly, a user's self-representation such as avatars may visualize the desired self instead of random pictures or personally relevant information, but again the software functionality remains the same for all users. In previous works, most of the attention in strong personalization research has focused on personalized content (see, e.g., Andrews, 2012; Cremonesi et al., 2012; Kaptein et al., 2012), but more than that could be offered (Klasnja et al., 2011).

We recognize here two issues with strong personalization which earn research interest and ways to mitigate them:

i. Going beyond mere offering personalized content by *personalized software features*.
ii. Addressing the oft-neglected fact in personalization efforts that also *users change over time*.

*Personalized software features* are more elaborate albeit more challenging approach than personalized content, and naturally some software features are more prone to strong personalization than others. For example, even though self-monitoring relates to an individual's measurements the key in it is not to convey the feeling that the software feature would be available “only for me”. Thus, data is personalized but the software feature is not since many other users will be able to conduct similar self-monitoring also. Virtual rehearsal is slightly different in this sense. When a user notices that a specific rehearsal suggested for them is unique rather only than selected from a list of options, it may influence the user to do the rehearsal. Similarly, virtual rewards have no intrinsic need for being personalized as there is no need for a user to think that no one else could get the same reward if they achieved the same results; in fact, it may be just the opposite, because social recognition may add to the power of virtual rewards. Also similarity and liking may benefit from a user's perception of strong personalization in modifying the way how information is being presented for the user. Thus, the key is whether a user's perception of using individualized software functionality instead of him or her feeling like a member of a target group plays a role or not.

Most personalization implementations today seem to assume that users stay the same over any given period of time, i.e., there is no modeling of *a user's possible change*. And yet, a user is more likely to change in multiple ways over any (longer) observation period. Similarly, persuasion profiles should not be static, but they could change over time, sometimes perhaps even with short notice. Moreover, a user may adopt certain roles or fulfill certain tasks that at times may require adopting a specific behavioral pattern, which may or may not be typical for the person. If the personalization engine in the software does not have the capability to recognize and process these types of inputs, any susceptibility model it produces may quickly become biased or even obsolete.

## Personalized Persuasive Software Features

In a digital intervention the content delivered, the timing of the intervention, as well as the interface used can be personalized to suit the needs and wants of a user (Berkovsky et al., 2012). Expanding from it, personalized software features involve the systematic use of individual user characteristics to determine relevant software features for the user. Personalization strategies may include adaptation, context awareness, and self-learning: Adaptation uses responses a user provides to questions to create personalized user experience, whereas context awareness senses and uses, for instance, the user's current location, location history, date, and/or time to create it, and self-learning uses new behavior data generated from the changing behavioral patterns of the user and system used to automatically adapt to the user's preferences (Monteiro-Guerra et al., 2020). Personalization by self-learning strategy is much more dynamic and requires developing much more complex algorithms than adaptation and context-awareness.

Some software features are more sensitive than others to provide opportunities for strong personalization. Persuasive software features in the Persuasive Systems Design model (Oinas-Kukkonen and Harjumaa, 2009) can be used to identify features that are sensitive to strong personalization. See Table 2. Features that enable strong personalization are mainly related to the computer-human dialogue support.

**Table 2:** Example of true personalized persuasive software features.

**Category**	**Software feature**	**Example implementation**
Computer-human dialogue support	Reminders	Personalized and/or customized reminders sent at an appropriate time using context-aware reminders
	Rewards	Personalizing rewards based on user preferences such as financial reward vs. cultural artifact
	Similarity	Using dialect or slang to represent the user's identity
	Liking	Personalized user interface based on a user's own style or taste
Primary task support	Virtual rehearsal	Personalizing a rehearsal based on user preferences and needs by recognizing user movement and proving the appropriate guide for a user to practice the desired movement

A system can offer personalized *reminders* by predicting opportune moments for sending notifications for a user or delivering reminders based on, e.g., a user's activity learned over time (Ghanvatkar et al., 2019). Such reminders provide unique functionality for a user because they are based on the appropriate times that are convenient for the user to engage in the desired activity. Reminders can also be designed to be customizable by the user. Enabling the user to customize a reminder is a means of prompting the user at their preferred time. However, there is the danger that timeslots chosen by the user aren't effective in terms of engaging in the desired activity; hence a context-aware reminder that learns the user's interaction behavior may perhaps be more effective (Singh and Varshney, 2019).

Generic *virtual rewards* tailored for users can be persuasive but there is also room to personalize rewards so that rewards could become even more relevant or appealing to the user. This can be done by using the user's preference of a reward, matching with the user's values, beliefs and culture, among other characteristics. Personalized rewards tend to be valuable to the receiver because they can be designed to be especially meaningful for her (Paay et al., 2015) and to reinforce the desired behavior by boosting a user's motivation and hence be persuasive (Li et al., 2021).

Users tend to prefer people or things that resemble themselves in some manner, such as in their values and previous experience; thus, *similarity* means that the system is analogous to the user in some way. For example, avatars can be created to resemble the user in the information system (Rheu et al., 2020), it is possible to mimic a user's living environment or culture (Li et al., 2021), or the self-representation of a user can be personalized using dialect or slang.

Personalized user interfaces (Nivethika et al., 2013) can make them more attractive and fit with a person's *liking* and subsequently enhance their persuasiveness. To enhance look and feel of a user interface, it is important to consider the depth to which it can be personalized. Forms of personalization can include user-enabled customizations and system-driven personalization (Bunt et al., 2007; Abdullah and Adnan, 2008), the latter of which can be based on user preferences or those aspects that the system learns from a user by means of machine learning algorithms. Context-awareness may help the system to adapt to the user's situation at hand (e.g., Lee and Choi, 2011).

In addition to the computer-human dialogue features of reminders, rewards, similarity, and liking, also primary task support by *virtual rehearsal* is sensitive to strong personalization. Personalized virtual rehearsals are relevant for behavior change interventions that require the user to learn a new desired behavior (Peng, 2009). Providing personalized behavioral rehearsals can support a user to practice the desired behavior (Langrial et al., 2014). For example, in the study by Clarke et al. (2020) the application encourages physical activity and guides the user to learn new physical movements and practice the desired behavior by adapting to the user's movements in a virtual rehearsal video and providing real-time feedback.

An important question is: at what point should a user notice that software is personalized in such a manner that the features and content are specific to his or her preferences, and what is the impact of this perceived personalization on user experience. This goes beyond simply claiming that software is personalized, which is usually the case for the one-fits-all type of systems when the extent of personalization is far from true personalization. In the same vein, the loose use of personalization as a marketing buzzword (Kim, 2002) is not advisable.

A major challenge with strong personalization naturally is having enough detailed information about the user. In the beginning of use there is typically not enough data to start personalizing the app; therefore some applications often run into the so-called *cold-start problem* (Banovic and Krumm, 2017). This can have an immediate impact on users who have high expectations for the system they are about to start using (Koch, 2002). To mitigate the cold-start problem and to meet the expectations of strong personalization, surveying questions about their preferences can be asked from users. Here again the level of personalization will be determined by the amount of information the user is willing to give about oneself to the app at a stage when they know so little about the app (Koch, 2002).

Some applications allow users to sign-up with user profiles from other platforms such as Facebook, Twitter, or LinkedIn. This may appeal particularly to users who like to maintain one profile across multiple applications (Karunanithi and Kiruthika, 2011); many of the user preferences have already been defined in that platform and they may be ready to be harnessed in other platforms, too. With the enforcement of the European Union's General Data Protection Regulation (GDPR) users own their data and can request a copy of their data, which may also contain information about their preferences in a format that can be, at least ideally speaking, imported into another application (Agyei and Oinas-Kukkonen, 2020). However, this often remains quite far from reality in practice.

Ideally, personalized interventions should perform better than one-size-fits-all interventions because they can better meet the specific and changing needs of a user (Rabbi et al., 2015a). In addition to that the extent of personalization in software may vary from weak to strong also the type of personalization may evolve over time. This can occur in two major ways. The system may adapt to the user preferences and directly influence the interaction, or the user interacts with the app in such manner that it changes the user model based on user's behavior (Zhu et al., 2021). If the system dynamically adapts to the user, then the level or spectrum of personalization offered by the system will vary at different points in time. To achieve the goal of strong personalization in information systems there is a need to constantly keep learning and adapting to users' changing needs and preferences. Designers should seek to ensure that true personalization really happens and that it is made clearly recognizable for users. By examining the software and its features and determining the extent to which they can and should be personalized on par with studying users' perception of such features may provide meaningful insights on the value of personalization as well as users' desire for true personalization.

## User's Change Over Time

A user of a behavior change support system may have had specific goals in mind when he or she started to use the system. However, these goals may change significantly or even become obsolete over time. There is also a high likelihood that something is going to interfere with the continued use of the system. A hectic situation in life may cause a temporary lapse in use, which in turn can lead the user even to stop interacting with the system altogether. Modern information systems can monitor users' interactions effectively and can perhaps even ascertain when these changes happen but are not very good at determining the reasons for it as it often requires direct feedback from the user. This lack of real-time and accurate predictive capability is one of the reasons why systems often do not correctly interpret the situation the user is in. Any interaction with the user who experiences a major change in life (for example, the person is hospitalized), can potentially result in miscommunication and thus may influence a decision to stop using the system.

It has been established that many users do not adhere to digital health interventions because of changes they face in their lives (Eysenbach, 2005; Karppinen et al., 2016; Lie et al., 2017). Eysenbach (2005) provides a list of hypothetical factors influencing non-usage and dropouts. For example, mundane reasons like lack of time are very common for not continuing in an intervention (Karppinen et al., 2016; Lie et al., 2017). Such lack of time is often due to difficulties participants face in their life that de-prioritizes the intervention. These events can vary a great deal, but it essentially means that there and then the use of the system is not very high on a user's priority list. The challenge for the support system is to handle these occurrences.

It is a common characteristic of digital health interventions to suffer from high dropout rates (Bremer et al., 2020). The number of dropouts tends to be heaviest within the first few weeks of the intervention (Eysenbach, 2005), and dropouts can often be attributed to attrition (Eysenbach, 2005). There have been a few advances in predicting dropouts. Pedersen et al. (2019) similarly observed that most dropouts happen early during the intervention, but they also identified three significant factors predicting dropouts: 2 weeks of inactivity in using the app, receiving less advice and engagement from the health coach, and quality of intervention program providers. Attrition happens over time, but the users tend to reduce their activity significantly a few weeks before the dropout (Pedersen et al., 2019). In many of these cases, it would be difficult to determine the real reasons without obtaining feedback from the user. User feedback is often provided reflectively when the period of use is over, but then it will be already too late to do anything about it. Thus, monitoring activity over time and responding to these lapses in a timely manner becomes the key to overcome the challenge (Pedersen et al., 2019). In their study of digital health interventions, Pedersen et al. (2019) were able to predict dropouts with 89 percent precision using the machine learning technique known as the random forest model (cf. Ho, 1995). This model has been used also in other fields, for example, to predict academic grades and academic dropouts (Rovira et al., 2017). A particular challenge that remains is to design systems that can reduce dropouts and improve adherence at the beginning of system's use when users typically value pragmatic aspects (Biduski et al., 2020).

There is also a temporal aspect related to intention to use and actual use of the system, namely a user perceives the system differently over time (Kujala et al., 2013). The temporality of user experience starts from anticipation and expectations of the interaction (Karapanos et al., 2009), and sequential process for user experience lifecycle can be defined (Pohlmeyer et al., 2010). Three main forces that are responsible for shifts in user's change have been described as familiarity, functional dependency, and emotional attachment (Karapanos et al., 2009).

Machine learning algorithms have potential to predict dropouts, changes in users' goals or preferences, and most suitable intervention types. Some behavior change support systems have demonstrated strategies which use personalized messaging, rotating interventions, and multi-armed bandits to gain feedback from users whilst trying out different intervention strategies (Paredes et al., 2014; Dempsey et al., 2015; Rabbi et al., 2015b; Kovacs et al., 2018). Rotating interventions seek to avoid the decline in effectiveness from static interventions by offering a multitude of different types of interventions where the support system acts more as a coach (Kovacs et al., 2018). With multi-armed bandits, the recommender system proposes different interventions for the user and learns directly from the feedback it receives (Paredes et al., 2014). Machine learning algorithms may ease the effort to understand user's changes on the individual level and respond to those changes accordingly.

Recommender systems provide an example of weak personalization in e-commerce is (Resnick and Varian, 1997; Lu et al., 2015). Sophisticated algorithms used by companies such as Netflix and Amazon are prolific at recommending products to people who use their services. On the one hand such algorithms have been developed to increase the profits and competitiveness of these companies, and on the other hand to increase user loyalty and retention over time. In reality, the weak personalization they offer does not care that much about the individual user and can even feel impersonal for the user.

**Figure 1:**
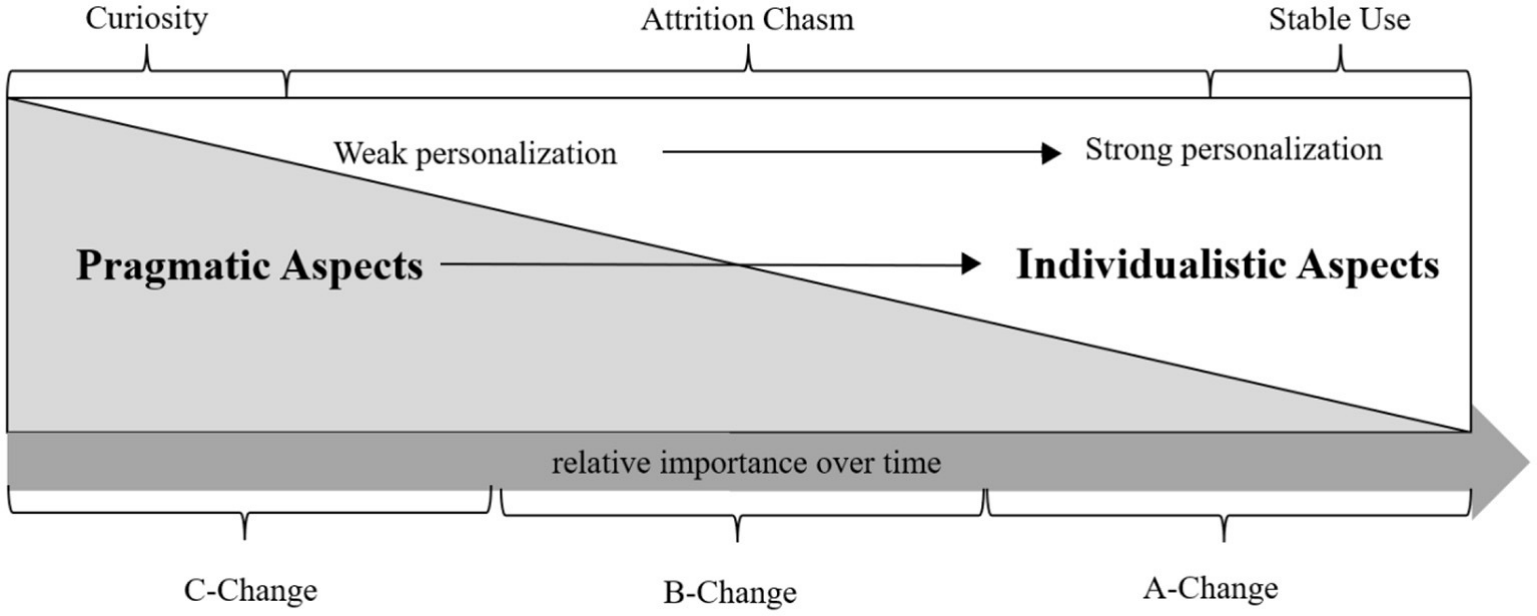
Crossing the attrition chasm.

Attrition chasm is defined as the point where the user stops using the system or at least the use of the system declines steeply over time; these phases were described by Eysenbach (2005) with recognition of high dropout rates from digital health trials after the curiosity phase. In Figure 1, we highlight the need to cross the attrition chasm to reach a more stable use phase. Biduski et al. (2020) further suggest that the user preferences change over time from general pragmatic aspects toward more individualized needs, where the user develops a deeper relationship with the system. We postulate that once the user gets past the curiosity phase of the experience, the benefits from true and from strong personalization increase relative to time spent with the system. Considering the behavior change types, A-Change regarding attitudes, B-Change related to behaviors and C-Change for compliance, research shows that it is considerably easier to make the user comply a few times during an intervention (C-Change), but to achieve attitude change (A-Change) takes a much longer time (Oinas-Kukkonen, 2013). If the desired outcome of the system is to bring about an A-Change, moving from weak to strong personalization is likely required, while not neglecting the psychological and emotional needs of the user during the overall process. In Figure 1, functional dependence starts with pragmatic qualities but continues to build over time along with familiarity, while the personalized qualities that cause emotional attachment are similarly built slowly and over time.

During the curiosity phase and shortly afterward, C-Change is more readily achievable. However, as time passes the user is likely to appreciate ways to individualize the system to fit their preference. In Figure 1, the types of behavior changes are presented to highlight the relative time required to achieve that type of change rather than that the change could not occur at an earlier or later stage depending on the individual. Notably, after the curiosity phase, the level of engagement comes to play, as the system builds a relationship with the user through meaningful interactions. This is when the system could learn a lot from the user. Nevertheless, Yardley et al. (2016) emphasize that more engagement with the user does not always mean that it is effective. This means that the system must be designed so that it is intelligent enough to adapt its level and timing of engagement.

Based on the three phases illustrated, Table 3 provides an example of true personalization strategies that can be used with a health behavior change support system. It should be noted that the curiosity phase can be relatively short while crossing the attrition chasm can take a longer time. As the duration of these phases may differ a lot between users and between systems, the goal should be to use the time effectively to learn and adapt to users' preferences and needs. In the example in Table 3, the system offers a variety of intervention mechanisms (content and software functionality) while employing machine learning algorithms not only to better predict user needs but also to improve the existing user profiles. Such a system can be made more adaptative to users by incorporating behavior change dimensions into the user profiles, for example, with the use of persuasion profiles (Kaptein et al., 2015). In addition, monitoring adherence and usage gaps in health interventions is vital from early on. This should continue throughout the life cycle of the intervention. While not possible to thwart all drop-outs, this personalization strategy should increase the adherence rate. Once the user reaches the stable use phase, the system should no longer rely on the *baseline* user profiles as it provides content and functionality based on the *individualized* user profile.

**Table 3:** Example of true personalization strategies for health behavior change support systems.

**Phase**	**Personalization strategy**	**Description**
Curiosity	The system provides tailored content and functions based on the *baseline* user profiles	User profiles based on initial setup such as questionnaire for personality traits or selected user preferences
	The system monitors gaps in use and engages the user when required	In health interventions, 2 weeks of inactivity has been found to be a predictor of a dropout
Attrition chasm	The system engages the users, monitors them, and learns about them by offering different content and functions, while occasionally asking for direct feedback	Use of machine learning algorithms such as rotating interventions, multi-armed bandits, or similar techniques, to build more accurate user profiles over time; persuasive software features can include different types of *suggestions and virtual rehearsals*
	The system dynamically interacts with the user by using a persuasion profile	Interactive tracking of user behavior while dynamically altering the content and functionalities users receive; this also improves the user profiles over time; can include persuasive software features such as *reminders, rewards, similarity*, and *liking*
Stable use	The system can provide the user with adapted individualized content and functionality	The system continues to learn about the user; offered content and functionality as well as level of engagement are based on *individualized* user profile

Users' needs, wants, goals and preferences often change during their time with a system, which further influences its usage patterns. For example, the experience of anticipating the use of a new system can positively or negatively result in the intention to use the system—or it can have very little effect on it. It depends on the anticipation and expectations that the user has toward the system, but also on their prior experience with similar systems (Karapanos et al., 2009; Biduski et al., 2020). The study from Biduski et al. (2020) indicates that it is important early on to build a positive image of the system and focus on more pragmatic aspects as mentioned earlier. In this, the focus should be on system qualities such as ease of use, usefulness, efficiency, convenience, and understandability, even if admittedly this can also vary between different kinds of users. Then over time, the relationship should be fostered with the user. It might be prudent to guide the user through the earlier experience for example by utilizing tunneling to support one to perform the primary task of the system.

Recognizing the user's personality can provide an additional layer of opportunities for strong personalization. The influence of personality traits demonstrates a clear effect but the degree can vary between different domains (Kaptein et al., 2015; Hales et al., 2017). For example, Su et al. (2020) found that young patients who are introverted but open to experience were both interested and willing to use a mobile diabetes application. In their study, conscientiousness that also reflects self-discipline did not play a significant role in the app use intentions. However, this result is inconsistent with another study on weight loss carried out by Hales et al. (2017) where self-discipline was found to be a significant factor. Su et al. (2020) argue that this discrepancy is because of population differences but it could also be because they are different types of applications. Su et al. (2020) also found out that open-mindedness was an important predictor of app use and adoption, and confirmed earlier findings that more conscientious users are likely to perform tasks more dutifully (Shambare, 2013). Yet, conscientious users may have difficulties adapting to new methods if they consider them as time-consuming or overly complex (Shambare, 2013; Su et al., 2020). Surprisingly, in spite of their attempt to investigate the role of emotional stability Su et al. (2020) didn't find that it to play a significant role in the acceptance of the app. Nevertheless, their conclusion was that understanding personality traits is important and can actually be used to predict who will continue to use the system in the longer span or to adhere to a health intervention.

Also user experiences develop and change over time and impact long-term use (Vermeeren et al., 2010; Kim et al., 2015). For instance, the use of a behavior change support system designed for a year-long weight loss journey typically starts with an active curiosity phase, but as time elapses so does the user activity. There is also a great deal of variance in the activity between users. Indeed, in most cases, users are far from being a homogenous group, and often those with less interaction with the system tend to have poorer outcomes (Karppinen et al., 2016). As time passes the relationship the user has with the system also changes, and those users who have developed a stronger and more meaningful relationship with it are likely to get a better user experience over time, which then results in an improved outcome for behavior change intervention (Karapanos et al., 2009; Biduski et al., 2020).

We suggest that it might not always be the best strategy to provide strong personalization in the early stages. At this time, a user may be overwhelmed with the amount of information as well as the amount of software functionality and navigational options the new system has to offer; basic design principles related to ease of use and providing guidance apply here, too. In later use periods, personalization may become much more evident when the user already has become accustomed to the system and familiar with its contents and features. Interactions can be further improved when the system knows the user better. It is essential to foster user loyalty by building a relationship between the user and the system. Therefore, true personalization may benefit from interactions instituted incrementally over time.

## Role of Artificial Intelligence

Artificial intelligence (AI) is an umbrella term that is a combination of techniques and methods for creating systems that can sense, reason, learn, act, and be used to solve problems (Rowe and Lester, 2020). AI systems can be made to perform a variety of tasks such as playing chess or intellectual tasks involving the use of some human elements of senses and reason. AI methodologies include big data analytics (to discover users' behavioral patterns patterns), machine learning (using algorithms to find patterns in data using supervised, unsupervised, semi-supervised, reinforced learning, and deep learning methods), natural language processing (capability of computers to process, analyze, and synthesize human languages), and cognitive computing (simulating human thinking processes and self-learning capability) (Chang, 2020). Table 4 outlines AI methodologies, their affordances for personalization, example applications, and ethical constraints.

**Table 4:** AI techniques, affordances, example application and ethical constraints.

**AI technique**	**Affordance**	**Example application**	**Ethical constraints**
Big data analytics	Collection and storage of massive amounts of different forms and kinds of user data to discover users' behavioral patterns (Hariri et al., 2019)	Identification of personalized risk factors to help people modify risks and prevent diseases (Barrett et al., 2013)	Privacy issues that emerge from collecting, processing, and using private information (Habegger et al., 2014)
Machine learning	Use of software algorithms to learn patterns from user data to make an intelligent decision and improve on the algorithm continuously (Sarker, 2021)	Individualized algorithms for predicting physical activity and providing interventions in real-time (Dijkhuis et al., 2018)	Validity of the classification and predictions (Valdivia et al., 2021)
Natural language processing	Automatic processing of human languages by computers (Lee et al., 2017)	Using natural language processing to understand user state and monitor user's emotional changes continuously and sensitively and give personalized feedback (Lee et al., 2017)	Privacy risk for processing sensitive data (Šuster et al., 2017) and ambiguity in representing and interpreting natural language (Chowdhary, 2020)
Cognitive computing	The ability of computer systems to simulate human cognitive processes, i.e., understand, reason, learn and interact (Behera et al., 2019)	The use of cognitive computing systems by bankers to analyze a vast amount of financial information including customer profiles to provide personalized wealth management advice to their customers (Hildesheim and Hildesheim, 2018)	Safety and performance-related issues as well as fairness (Behera et al., 2022)

AI can drive the development of truly personalized systems. For example, this can involve effecting behavior change by enhancing the efficiency of self-monitoring (Chew et al., 2021). Data can be collected and used to optimize goal setting and action planning (building and validating personalized predictive models), and provide personalized micro-interventions (e.g., prompts, nudges, and suggestions) in real-time to achieve the desired goal (Chew et al., 2021). Personalization enables system-tailored content and functionality to be offered to users based on their characteristics, needs, and preferences. Such systems can produce rich data about the user, process the data, provide insights, and support the user's ongoing activities. For instance, just-in-time systems (Intille et al., 2003) employ decision rules which use the current state of the user (e.g., emotional state and environmental conditions) as input to choose the time and type of intervention to deliver to the user (Menictas et al., 2019).

**Figure 2:**
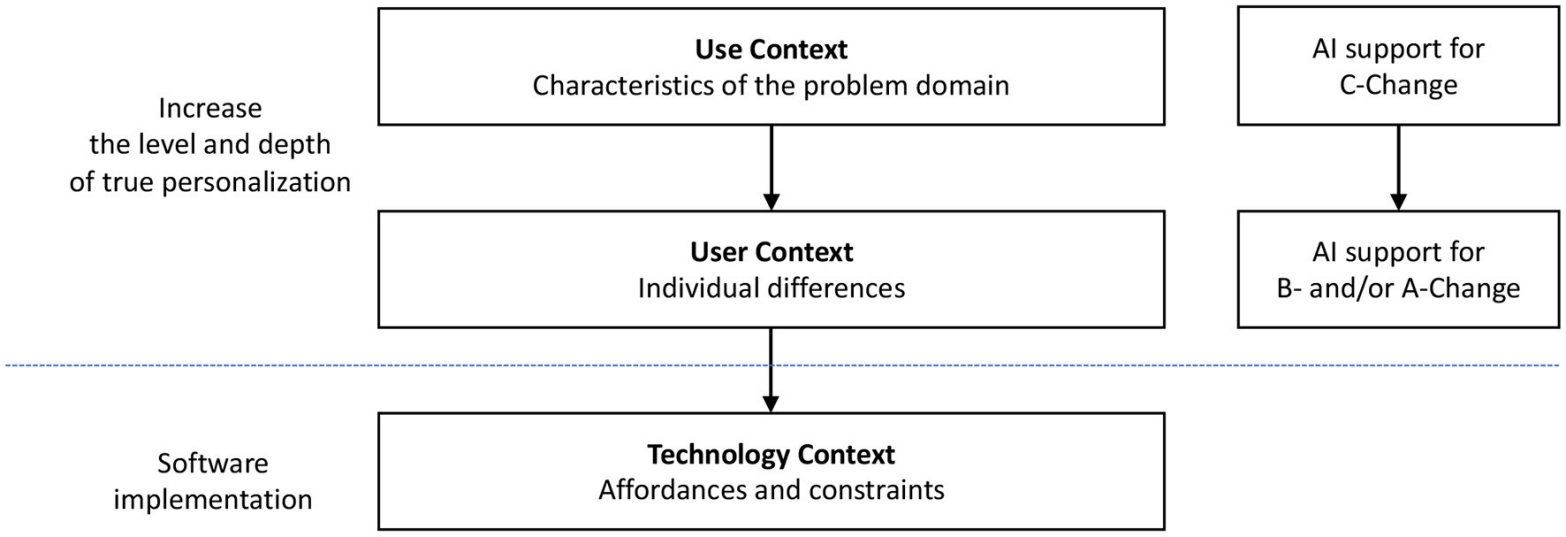
Conceptual AI framework for developing strong personalization.

To elaborate on the move toward a strong personalization strategy, developers need to aim beyond user segments by designing and developing systems that recognize individual differences and preferences. Figure 2 provides a framework that describes how such personalization can be achieved. Availability of both technology and related skills need to be considered when utilizing artificial intelligence. It is crucial to carefully consider the type of AI needed to improve personalization. For example, tailoring might be good enough for weak personalization and suitable for systems that intend to affect a compliance type of change (C-Change). This is because the aim is for users simply to comply with the requests of the system. Very limited AI capability may well be enough to achieve this effect. Strong personalization, to help realize B-Change or A-Change, may require much more complex AI mechanisms that can model the user characteristics, preferences, changing needs, etc., and often in real-time.

In developing personalized systems powered by AI, ethical challenges can emerge from the Use Context (including laws and regulations in specific problem domains such as health, finance, or retail), User Context (including privacy, safety, and preferences), or Technology Context (including security and technological limitations) or any combination of these. Although AI can increase the feasibility of developing truly personalized systems, there are ethical issues that developers grapple with. These include, but are not limited to, transparency (Yu and Alì, 2019), privacy (Chowdhary, 2020), safety and performance (Behera et al., 2022), accessibility to artificial intelligence (Morris, 2020), moral agency (Swanepoel, 2021), biases stemming from data collection process, data, and algorithm (Li et al., 2019), threats to human autonomy (Sankaran et al., 2020), environmental and ecological challenges arising from hardware and energy consumptions required to develop and operate AI technologies (Li et al., 2019), liability issues in event of a damage or harm (Cerka et al., 2015), and social acceptability (Morris, 2020).

The ethical issues in AI technologies are caused by limitations in the technology itself [e.g., the use of black-box algorithms such as deep learning, random forest, support vector machines which are less explainable but provide high accuracy precision (Chang, 2020)], insufficiencies in AI regulations and policy, and insufficiency of existing ethical design principles (Li et al., 2019). Also, true personalization requires detailed information about the user and hence users must be willing to reveal personal information to benefit from it (Kobsa, 2002). This personal information needs to be collected, analyzed, and interpreted so that truly personalized user experiences can be provided. For this process, many data management, sharing, and privacy requirements should be considered and adhered to. These ethical issues make it critical to obtain informed consent from the user by providing clear and adequate information (Agyei and Oinas-Kukkonen, 2020; The European Commission, 2021). Indeed, there is all the reason to believe that AI methods will be used in the future to a very large extent to enhance the personalization. From an ethical perspective, it is important to be transparent to the user about the intention of the AI system in use while also not collecting information about the user that is not relevant to the operation of the system.

## Conclusion and Discussion

In the era when there is so much discussion around personalized systems and technologies, this article sought to raise interest and discuss regarding Personalization Myopia and the nature of true personalization. Even with a plethora of academic research into personalization in general, strong personalization is yet to gain notable momentum. Personalizing software functionality is much more complex than personalizing contents *via*, e.g., feedback, simulation (Mcalpine and Flatla, 2016), or social comparison (Zhu et al., 2021). Thus, “personal” and “personalized” are different concepts.

Users also change over time. This means that also the grounds for personalization may change without the system noticing it, thus leading to the system having an outdated view of the user. We posited here that trying to make a system personalized from the very beginning of usage might be even harmful, but rather the depth of personalization could be increased over time. It remains a challenge, how a change in behavior can be reliably detected and measured. In addition, how can a change in a user's goal be detected and determined, if the user is not provided with an explicit goal-setting feature? Furthermore, technological platforms upon which the information system has been built may also change.

Yet, whether strong or weak personalization, some kind of user profiles are needed. With users being profiled, the need for privacy and acknowledgment of laws and regulations (e.g., GDPR and Medical Device Regulation in the European Union) related to it play a critically important role in the development of applications. In practice, weak personalization in many cases may be a more desirable approach as it is likely to require a lesser amount of data from the users on an individual level. We would also like to see more contributions to the scientific discourse around theory vs. design-driven approaches (Arriaga et al., 2013).

There remains many research challenges. The user's emotions impacts both user experience and continuance intention, but the relationship between user emotions and personalization is not well understood and should be explored in more detail for instance with regards to health behavior change. For another matter, support systems in the health domain often require continuous use and adherence to fulfill their purpose. Other open questions are many, too: What kind of general claims can be made of perceived user experiences if each user is offered a different software entity? Does customization lead to the Do-Your-Own-System dilemma? Information systems with their endeavor to provide a user with fitting, useful, and/or influential information at the same time may also be filtering out information that actually could be highly relevant or perhaps even critical for the user; thus, personalized solutions are predisposed to filter paradox (Oinas-Kukkonen and Oinas-Kukkonen, 2013, p. 84). Furthermore, there may be underprivileged user groups such as the elderly who might have little say or perhaps no understanding at all about the downside of what such filtering would mean in practice. Yet another challenging question is whether personalization kills exploration? Such questions earn more attention in future research.

## Data Availability Statement

The original contributions presented in the study are included in the article/supplementary material, further inquiries can be directed to the corresponding author/s.

## Author Contributions

HO-K: conceptualization, methodology, funding acquisition, resources, and supervision. HO-K, SP, and EA: writing—original draft preparation and review and editing. All authors have read and agreed to the published version of the manuscript.

## Conflict of Interest

The authors declare that the research was conducted in the absence of any commercial or financial relationships that could be construed as a potential conflict of interest.

## Publisher's Note

All claims expressed in this article are solely those of the authors and do not necessarily represent those of their affiliated organizations, or those of the publisher, the editors and the reviewers. Any product that may be evaluated in this article, or claim that may be made by its manufacturer, is not guaranteed or endorsed by the publisher.
